# High Critical Current Density of YBa_2_Cu_3_O_7−x_ Superconducting Films Prepared through a DUV-assisted Solution Deposition Process

**DOI:** 10.1038/srep38257

**Published:** 2016-12-01

**Authors:** Yuanqing Chen, Weibai Bian, Wenhuan Huang, Xinni Tang, Gaoyang Zhao, Lingwei Li, Na Li, Wen Huo, Jiqiang Jia, Caiyin You

**Affiliations:** 1 School of Materials Science and Technology, Xi’an University of Technology, Xi’an, Shaanxi 710048, China; 2 College of Chemistry & Chemical Engineering, Shaanxi University of Science & Technology, Xi’an, Shaanxi, 710021, China

## Abstract

Although the solution deposition of YBa_2_Cu_3_O_7−x_ (YBCO) superconducting films is cost effective and capable of large-scale production, further improvements in their superconductivity are necessary. In this study, a deep UV (DUV) irradiation technique combined with a low-fluorine solution process was developed to prepare YBCO films. An acrylic acidic group as the chelating agent was used in the precursor solution. The acrylic acidic group was highly sensitive to DUV light at 254 nm and significantly absorbed UV light. The coated gel films exposed to DUV light decomposed at 150 °C and copper aggregation was prevented. The UV irradiation promoted the removal of the carbon residue and other by-products in the films, increased the density and enhanced the crystallinity and superconductivity of the YBCO films. Using a solution with F/Ba = 2, YBCO films with thicknesses of 260 nm and enhanced critical current densities of nearly 8 MA/cm^2^ were produced on the LaAlO_3_ (LAO) substrates.

YBCO films or YBCO coated conductors with high critical current densities (J_c_) are promising in applications for power transportation and electronic or electrical devices. Solution depositions of YBCO films are commonly studied because they are cost effective and capable of large-scale production^1^,^2^. YBCO films with a high J_c_ above 1 MA/cm^2^ are typically produced through a solution process using trifluoroacetates (TFA) as precursors. During the TFA solution process, the coated gel films were pyrolyzed to form precursor films, and then the precursor films were annealed and post annealed to produce the YBCO films^3^,^4^.

Controlling the pyrolysis process was essential to ensure the quality of the YBCO films. During the pyrolysis process, the gel films were heated and the organic species in the gel film decomposed, which released gases from films and formed a porous precursor film. The porous structure is one characteristic of a solution processed inorganic films, and is partially responsible for the inferiority of electrical properties in solution processed oxide electronic films compared to those deposited by physical vapor deposition methods such as magnetic sputtering and pulse laser deposition. The pyrolysis process of YBCO gel films was also reported to cause the copper organic species to easily sublime^5^. As such, the copper element was commonly lost or not uniformly distributed inside the precursor film^5^,^6^. The copper also aggregated on the film surface after the Cu-salts decomposed during the pyrolysis process, which induced second phases inside the final annealed YBCO films. Both problems synergistically degraded the quality of solution-processed YBCO films.

Recently, a solution process combined with deep ultraviolet (DUV) irradiation was reported, and high-performance oxide films were produced by Kim *et al*.^7^. In this process, high-energy photons emitted from a DUV-lamp penetrated into the gel films and triggered radical-forming reactions which broke the chemical bonds^7^,^8^,^9^,^10^. Obviously, the photo-activation process is completely different from the traditional thermal initiation process. Gel films exposed to a DUV light decomposed at low temperatures. For example, Park *et al*. used the DUV irradiation method to fabricate metal oxide films on a flexible plastic substrate by the sol-gel method^8^. Studies indicated that during UV irradiation, film densification occurred from the removal of impurities such as decomposed metal ligands and other by-products. The DUV irradiation was also reported to effectively prevent the loss of some elements. Bretos *et al*. reported that Pb volatilization was minimized when UV light was used to irradiate the Pb_0.76_Ca_0.24_TiO_3_ film^11^. Their work also confirmed that the UV-irradiated film was denser than films prepared without UV irradiation. From these results, we hypothesized that if UV irradiation was used to prepare the YBCO films, the Cu aggregation could be prevented and the films would be denser since the photo-activation process occurred at a low temperature, and therefore YBCO films could be produced with enhanced J_c_.

In this study, we prepared YBCO films using a novel precursor solution sensitive to 254 nm of UV light. The precursor films were produced at 150 °C with assistance from DUV irradiation, as illustrated in Figure S1. No other pyrolysis processes typically used in the fabrication of YBCO films were necessary. After the irradiated film was annealed at 785 °C, YBCO films were produced with a high J_c_ of nearly 8 MA/cm^2^. Finally, we studied the photochemistry during UV irradiation, the formation of the intermediate phases and the influence of UV irradiation on the chemical and physical properties of the films.

## Results and Discussion

**Figure 1:**
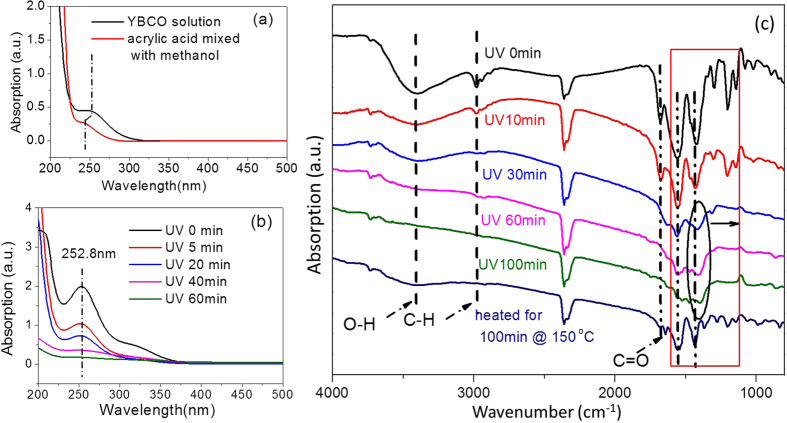
(**a**) UV-visible absorption spectra of the YBCO solution and a mixture solution of acrylic acid and methanol; (**b**) UV-visible spectra of dried YBCO precursor films irradiated under DUV light for different periods of time; and (**c**) FT-IR spectra of YBCO gel films irradiated for different periods of time at 150 °C under the DUV lamp.

Figure 1(a) shows the UV-visible spectra of an YBCO precursor solution and a mixture solution of acrylic acid and methanol. For the mixture solution of acrylic acid and methanol, an absorption peak was located at 248 nm. This peak was assigned to the characteristic absorption of the acrylic acid. However, for the YBCO solution prepared with acrylic acid, this peak shifted to 252.8 nm. This shift indicated that the Y, Ba, Cu ions reacted with the acrylic acid, forming the corresponding chelating ligands. In our experiments, UV light with wavelength of 253.7 (90%) and 184.9 nm (10%) was emitted from the mercury lamp. Thus, the absorption peak at 252.8 nm of the gel films derived from the YBCO precursor solution was highly sensitive to the 253.7 nm DUV light. Figure 1(b) shows the UV-visible spectra of the YBCO gel films irradiated for different period of times. When the time of irradiation increased, the peak intensities of the UV absorption spectra of the YBCO gel film gradually decreased and were non-existent after 60 minutes of irradiation. As such, the structures of the metallic salts chelated with the acrylic acid were destroyed after irradiation.

The decomposition of the organic species in the gel films was also examined using the FT-IR spectra analysis. It was well known that different absorption peaks in the FT-IR spectra correspond to the vibrations of different organic species. According to Fig. 1(c), the O-H (3405 cm^−1^), C-H (2890 cm^−1^) and C = O (1680 cm^−1^) stretching vibration bands gradually decreased as the irradiation time increased. The characteristic peaks (1563 cm^−1^, 1429 cm^−1^, 1295 cm^−1^, 1196 cm^−1^) enclosed by the red rectangle in Fig. 1(c) were attributed to the bending or stretching vibrations of C-C, C-H and C-O, which nearly disappeared after 100 minutes of irradiation^12^,^13^,^14^,^15^,^16^. Interestingly, as seen from the spectra of the gel films irradiated for 30, 60 and 100 minutes, the peaks located at wavenumber 1429 cm^−1^ gradually diminished and shifted slightly towards the lower wavenumber, as indicated by the black circle. This was related to the graphite-like carbon or disordered carbon formed inside the films^14^,^15^,^17^. In contrast, for the non-UV irradiated films, characteristic peaks of the organic species were still observed after being dried at 150 °C for 100 minutes. These results implied that the high-energy DUV photons effectively caused the organic compounds in the gel films to decompose.

**Figure 2:**
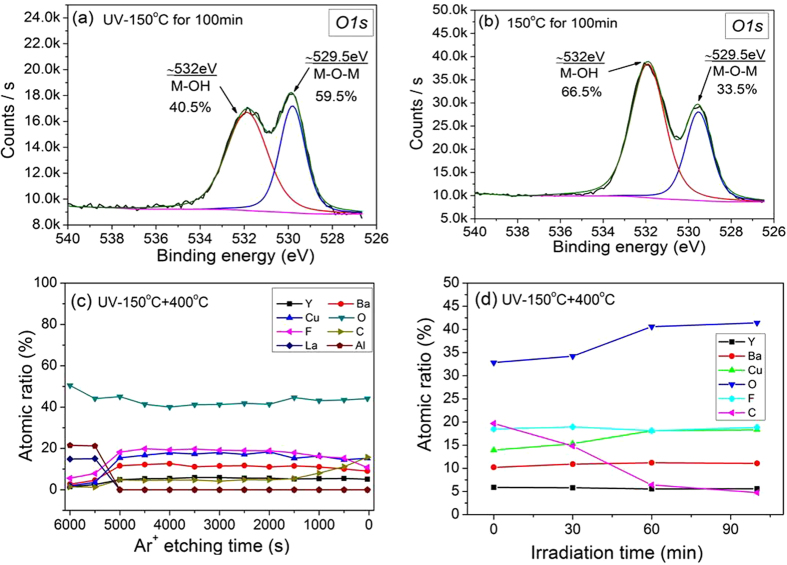
XPS spectra of O(1 s) spectra for (**a**) the UV-irradiated sample and (**b**) the non-UV irradiated sample. Both samples were heated to 400 °C under a nitrogen atmosphere with 500 ppm O_2_. The data of the O1s spectra was collected after 2000 seconds of etching. (**c**) the depth profile of a sample UV-irradiated for 100 minutes. (**d**) the atomic ratio of elements in the samples irradiated under UV light for different amounts of time.

Since the organic species of the gel films were, at least partially, decomposed by the DUV light, the noncrystalline or nanocrystal metallic salts may have formed in the films. An XPS investigation was conducted on the samples, and the O1s peaks are shown in Fig. 2. In Fig. 2(a) and (b), two O1s peaks are centered at 529.5 eV and 532 eV, respectively. The binding energy (BE) of 529.5 eV was related to the M-O bonding of the oxygen atoms in the lattice. The BE of 532 eV is typically attributed to the oxygen atoms in M-OH compounds^7^,^8^. Figure 2(a) and (b) show that for the UV irradiated films, the oxygen in the lattice totaled 59.5%. However, for the non-UV irradiated films, the oxygen in lattice was only 33.5%, which confirmed that the high-energy DUV photons induced photochemical cleavage of some organic groups, and activated metal and oxygen atoms to facilitate the formation of the M-O-M network. It should be noted that after the films decomposed, the oxide in the films primarily contained CuO because the Ba or Y ions formed in the Ba-Y-F, BaOF, or YOF phases, as discussed later in this paper. As such, the M-O primarily referred to the Cu-O bonds.

After UV irradiation, the film was heated to 785 °C under humidified N_2_ gas with O_2_ of 500 ppm to obtain crystalline YBCO films. To further investigate the effects of UV irradiation on the films, the irradiated films were quenched at different temperatures (400 °C, 525 °C, 650 °C and 785 °C) during the heating process, and then XPS and XRD analyses were conducted. To determine the distribution of the elements, an XPS depth profiling was performed on the films quenched at 400 °C. The depth profiling was performed using an Ar^+^ bombardment. For the irradiated film quenched at 400 °C (at which temperature the sample was held for 15 minutes), the Y, Ba, Cu elements were uniformly distributed in the UV irradiated film, as shown in Fig. 2(c). Cu accounted for 16~19%, and Y and Ba accounted for 5~6% and 10~12%. These percentages were close to the ratio of Y:Ba:Cu = 1:2:3 in the solution. An XPS depth profiling was also performed on the non-UV irradiated sample, and the reference sample was prepared using the traditional pyrolysis process. For the non-UV irradiation sample, the distribution of the copper content along the direction of the film thickness was uneven. The Cu element aggregated on the film surface. Inside the film, the amount of Cu in some areas was even less than that of the Ba element, as shown in Figure S2(a). The reference sample had nearly the same results with observable Cu aggregation, as shown in Figure S3(a).

The Cu aggregation on the film surface during the traditional thermal pyrolysis process may be resulted from the sublimation of the Cu organic species at high temperatures^6^. However, as mentioned above, the UV irradiation made the Cu organic species decompose at a temperature lower than its sublimation temperature, and boosted the formation of the Cu-O bonds inside the film. Therefore, the loss or aggregation of Cu was prevented, which led to the uniform distribution of metallic ions along the film thickness and promoted the growth of phase-pure YBCO films. The elemental distribution was also analyzed on the films UV irradiated for different times, as shown in Fig. 2(d). The Y, Ba, Cu elements maintained a constant ratio of 1:2:3 after being irradiated for 60 minutes. However, for films irradiated less than 60 minutes, Cu was lost to some degree. Moreover, during the first 60 minutes of irradiation, the carbon content rapidly decreased, which indicated that the film condensation primarily occurred during the first 60 minutes.

**Figure 3:**
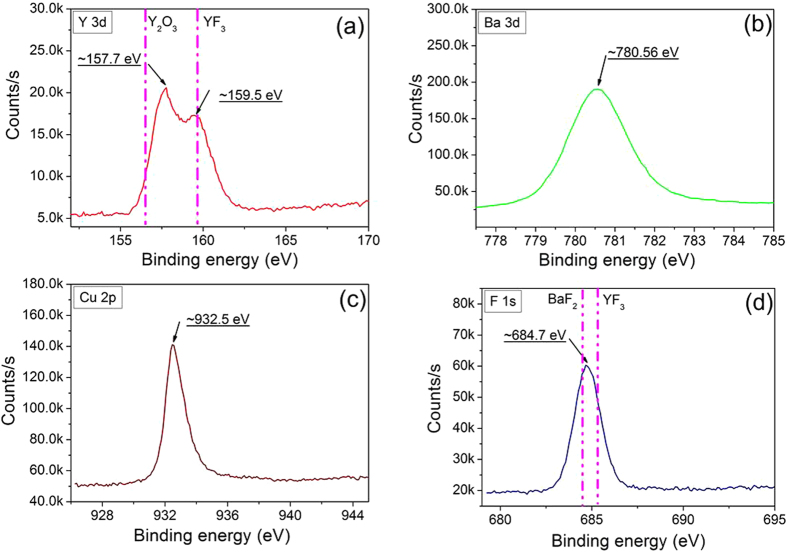
The XPS spectra of Y3d, Ba2d, Cu2p and F1s for the UV irradiated samples quenched at 400 °C.

Figure 3 shows the XPS of Y, Ba, Cu, F in the UV irradiated sample after being heated to 400 °C. The Y3d spectrum depicted a double structure associated to the spin-orbit split with two intense peaks located at 157.7 eV and 159.5 eV. Y3d_5/2_ components in YF_3_ and Y_2_O_3_ are typically at BEs from 159~160 eV and 156.5~157 eV^18^,^19^, therefore, small amounts of YF_3_ in the films were predicted. However, the Y_2_O_3_ can be excluded. The strong peak located at 157.7 eV was the phase of the YOF clusters. The Ba3d _5/2_ spectrum displayed a peak at 780.6 eV which was BaF_2_ with the BE previously reported at 779.8 eV^20^. However, these values were close the Ba BE for the BaO and BaCO_3_^20^,^21^. The Cu 2p core level had a peak at 932.5 eV, which was assigned to the CuO or Cu_2_O phase^22^. The BE of F1s was centered at 684.7 eV, which was associated with the possible phases of the Y, Ba, and Cu fluorides. The CuF_2_ was excluded from the Cu 2p spectrum^23^. F1s components in BaF_2_ and YF_3_ were typically located at the BEs of 683.7 eV and 685.3 eV^24^. Therefore, the BaF_2_, YF_3_, BaOF, and YOF were the main phases in the films, in addition to the CuO or Cu_2_O phase.

**Figure 4:**
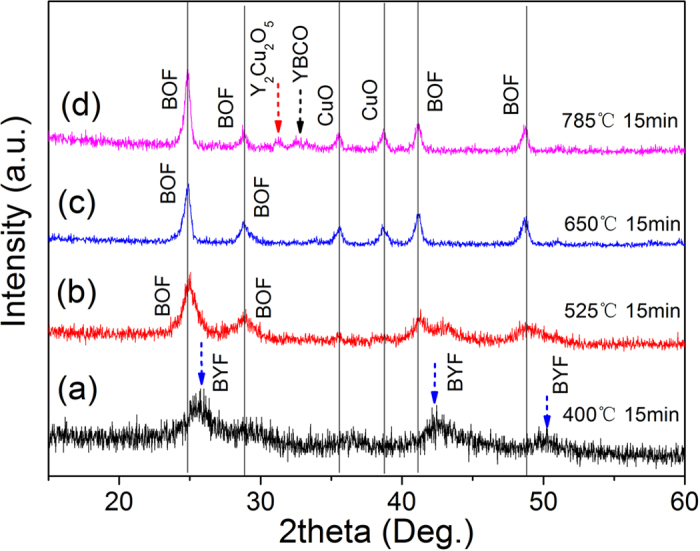
Grazing-incidence XRD patterns of the films quenched at different temperatures.

To further verify the phase structures, a grazing-incidence X-ray diffraction was performed on the samples, as shown in Fig. 4. The film heated to 400 °C had broad peak at 25.7° and 42.5°. Neither peak corresponded to the Ba or Y fluorides, but was considered a signature of the BaF_2_-YF_3_ solid solution (Ba_1−x_Y_x_F_2+x_, BYF). The calculated lattice parameter of the face centered cubic BYF was 0.61 nm. The cubic BaF_2_ had a lattice parameter of 0.62 nm^25^. Therefore, the increase of the YF_3_ concentration in the BaF_2_ lattice induced a decrease of the lattice parameter. Based on the reported experimental dependence of unit cell parameters on the Y content^26^, x~0.24 can be estimated. It should be noted that F/Ba was 1.6~1.8 in the 400 °C precursor film according to the XPS results. Therefore, in addition to BYF phase, BaOF and YOF also existed in the films, which confirmed the data from the XPS. However, these intermediate phases were different from the precursor film obtained by the traditional pyrolysis process (reference sample). When the F/Ba = 2 solution was used, only the BaF_2_ or BaOF were detected in the reference sample, as shown in Figure S3(b). After the irradiated films were heated to 525 °C, the Ba^2+^ ions were in the form of BaOF/BaF_2_ (F/Ba < 2) in the film, since only the diffraction peaks of 24.9° were detected. This indicated that the BYF decomposed to BaOF/BaF_2_ and Y_2_O_3_ after the film was heated to 525 °C. At 785 °C, the Y_2_Cu_2_O_5_ and YBCO phases were detected, which were formed by the Y_2_O_3_ and CuO, BaOF/BaF_2_, and water gas. All reaction paths during the 525–785 °C were the same as those in traditional TFA routes^27^.

**Figure 5:**
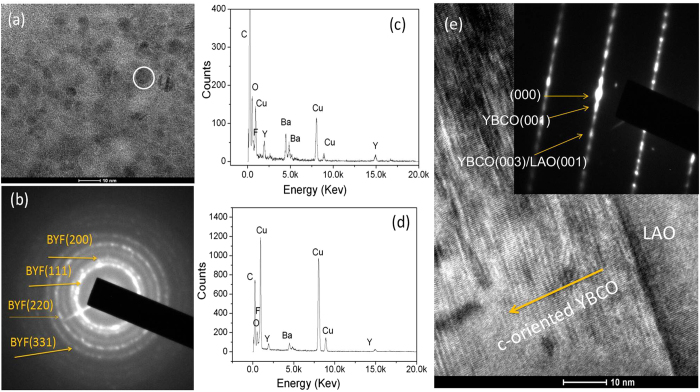
(**a**) and (**b**) are the cross-sectional TEM image and SAED pattern of the precursor film. The precursor film was processed by UV irradiation for 100 minutes and heated to 400 °C. Some ‘dark’ spherical particles were observed to be embedded in the crystalline matrix. The EDS image of the entire precursor film is shown in (**c**), and (**d**) is the EDS map of a ‘dark’ spherical particle circled in (**a**). The HRTEM image and SAED pattern of the annealed YBCO film that was UV-irradiated for 100 minutes on the LAO substrate are shown in (**e**).

TEM was conducted to determine the morphology of the precursor film and the final crystallized YBCO film. Figure 5(a) shows the HRTEM morphology of a precursor film UV-irradiated for 100 minutes and heated to 400 °C. A selected area electron diffraction (SAED) as shown in Fig. 5(b) indicated that the BYF phase formed in the film, consistent with the XRD results (Fig. 4(a)). The elements Y, Ba, Cu, O and F were detected in the precursor film (Fig. 5(c)), which indicated that the metallic ions of Y, Ba and Cu combined with F and O to form corresponding fluorides and oxides, as confirmed by the XRD results in Fig. 4(a). Some ‘dark’ spherical particles were observed to be uniformly embedded in the BYF matrix. The EDS map (Fig. 5(d)) indicated that these ‘dark’ spherical particles were CuO particles. The sizes of the CuO particles were generally less than 5 nm. After the films were annealed at 785 °C, and epitaxial YBCO films formed on the LAO substrate. Figure 5(e) shows the HRTEM image and the SAED pattern of a YBCO film UV irradiated for 100 minutes on the LAO substrate. The film was epitaxially grown on the LAO substrate along the c-axis orientation, with no interdiffusion between YBCO and the LAO substrate.

**Figure 6:**
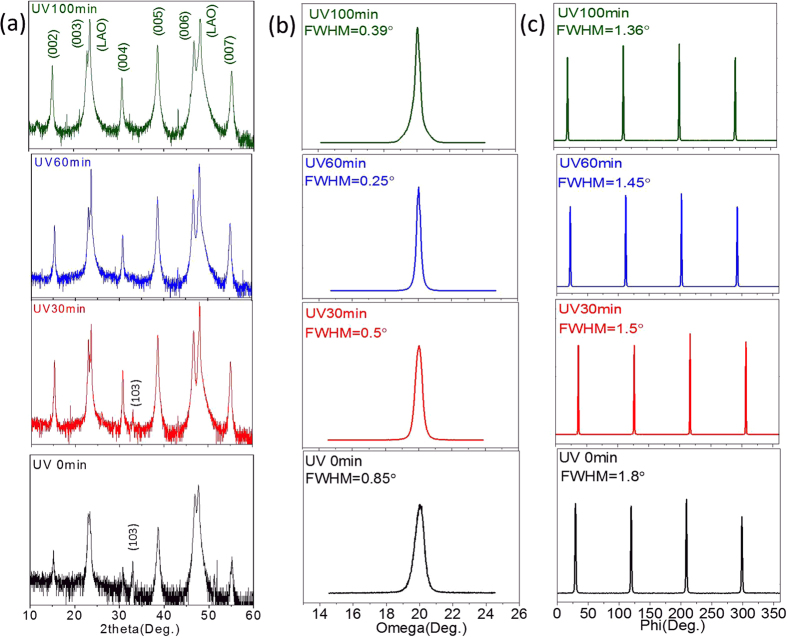
The XRD patterns of different samples: (**a**) is the theta-2theta diffractions of YBCO films irradiated by UV light for different times, and (**b**) and (**c**) are the omega and phi scanning of corresponding films.

The high crystallization and pure c-axis orientation of the YBCO grains were two important factors for the high superconductivity of YBCO films. The orientation and phase structures of the YBCO films were detected by the XRD and displayed in Fig. 6. For the non-UV irradiated film, (103)-oriented grains and (200)-oriented grains were observed in their theta-2theta XRD patterns (Fig. 6(a)). Only (00 ***l***)-oriented grains were detected in the UV irradiated films with irradiation times over 60 minutes. These results were related to the intermediate phases inside the films. As mentioned above, in the irradiated film, the Ba^2+^ existed as BaF_2_ and BaOF before the formation of the YBCO phase, which was favorable for the growth of the c-oriented YBCO grains. However, inside the precursor films that were not UV irradiated, BaOF/BaF_2_, and BaCO_3_ were detected, as revealed by XPS as shown in Figure S2(b). The BaCO_3_ phase was reported to be one of the obstacles preventing the formation of pure YBCO phases. In addition, the Cu element was not uniformly distributed inside its precursor film, which also degraded the film crystallinity. As shown in Fig. 6(a), the diffraction peaks of the (103) plane were completely eliminated after 60 minutes of UV irradiation. And the intensity of diffraction peaks of (005) and (006) planes increased with irradiation times. The film textures were examined by a phi scanning of the (103) plane and an omega scanning of the (005) plane using a high-resolution X-ray diffractometer, as indicated by Fig. 5(b) and (c). We found that the full width of the half maximum (FWHM) for both the phi scanning and the omega scanning decreased with UV irradiation times, indicating that the in-plane and out-of-plane textures of the YBCO films improved.

**Figure 7:**
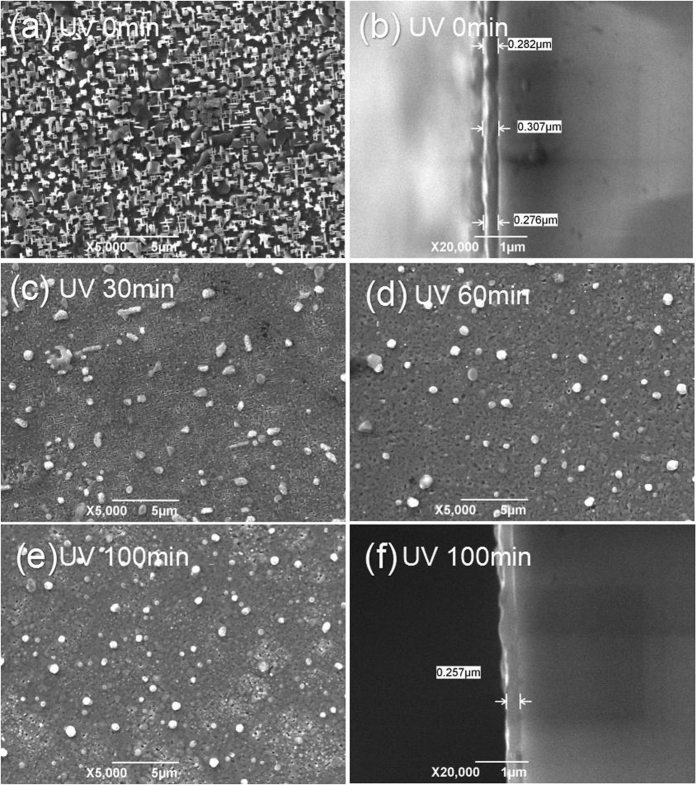
Surface morphology and cross-sectional morphology of different YBCO films: (**a,b**) the non-UV irradiated film; (**c–f**) the UV-irradiated films.

The film surfaces and cross-sectional morphologies were examined by SEM, as indicated in Fig. 7. The non-UV irradiated film annealed at 785 °C showed an undesired surface morphology. Some randomly oriented grains and a-axis oriented grains were observed on the film surface, consistent with the results of the XRD shown in Fig. 6(a). By contrast, the UV-irradiated films had a dense structure and no apparent pores, as displayed in Fig. 7(c–e). The thickness of UV-irradiated films (250 nm) was lower than the non-UV irradiated samples (280 nm), indicating that the film density improved, which was consistent with the enhanced intensity of the diffraction peaks of (00 l) planes determined by XRD. The improved film density was caused by several factors. First, during UV-irradiation, undesired by-products such as C and H elements were removed by reactions with radical oxygen^16^,^17^, which made the film denser than the films without DUV irradiation. Second, the increased film density was related to decomposition kinetics. Llordes *et al*. reported that porosity could be reduced by lowering the pyrolysis temperature^5^. In our experiments, the gel film decomposed at 150 °C under UV irradiation, more than 200 °C lower than decomposition during the traditional pyrolysis process. Finally, the improved film density was related to the new processing method developed in this study. Here, the gel films were UV-irradiated and then heated under nitrogen gas with 500 ppm O_2_. It is reported that the porosity of the 400 °C-pyrolyzed precursor film could be reduced to 5% when heated under a less-oxidized atmosphere. However, for the film pyrolyzed under oxygen gas, the porosity was as high as 30%^5^.

In addition to the film density, it should be noted that some white particles were observed on the film surface of all the samples. These white particles were Cu-rich phases and were related to our new processing method. After the gel film was irradiated, it was directly annealed under nitrogen with a small amount of oxygen. Under atmospheres with low oxygen pressure, coarse CuO/Cu_2_O particles were observed on the film surface by Llordes *et al*.^5^. Our experiments confirmed their results, especially for the non-UV irradiated film. Fortunately, the UV irradiation suppressed the ripening of the Cu_2_O/CuO particles. As demonstrated by the SEM figures, when the UV irradiation time increases, the size of the white Cu-rich particles decreases. This also explains why the Cu element is uniformly distributed inside the UV-irradiated precursor film, as mentioned above.

**Figure 8:**
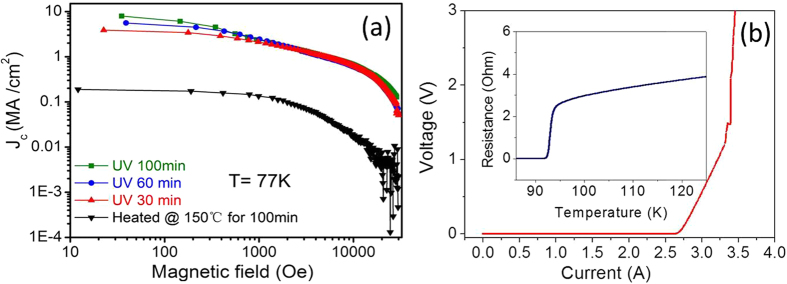
(**a**) shows the J_c_-H curves of a non-UV irradiated YBCO film and UV-irradiated films, and (**b**) presents the I-V curve and R-T curve of a YBCO film irradiated for 100 minutes.

Figure 8(a) presents the J_c_-H curves of the YBCO films. The J_c_ value was calculated according to the Bean Model. The film without UV irradiation only showed a low J_c_ of 0.19 MA/cm^2^ at 77 K, 0 T, which was attributed to the Cu-aggregation, and the existence of a-axis grains on the film surface (shown in Fig. 7(a)) resulted from the poorly controlled nucleation. The formation of the a-axis grains also led to some porosity and further decreased the self-field J_c_^2^,^28^. However, the J_c_ values of films UV irradiated for 30, 60 and 100 minutes reached high values of 3.88 MA/cm^2^, 5.58 MA/cm^2^ and 7.95 MA/cm^2^ at 77 K, 0 T, respectively. The film irradiated for 100 minutes was patterned to be a micro-bridge with a width of 150 μm. Its transport voltage–current curve (V-I curve) is shown in Fig. 8(b). The critical current of the micro-bridge was 2.66 A with a criterion of 1 μV/cm, which corresponded to the critical current density J_c_ of 6.8 MA/cm^2^ (77 K, 0 T), close to the value calculated by the Bean Model. The insert of Fig. 8(b) shows the R-T curve of the film irradiated for 100 minutes, and the critical transition temperature was approximately 92 K. The J_c_ of UV-irradiated YBCO films increased with irradiation times, since the prolonging of irradiation times promoted the decomposition of organic compounds, enhanced the removal of the carbon residue, and effectively avoided the copper segregation in the precursor films. In terms of YBCO films prepared using conventional pyrolysis method, the best J_c_ values were 5 MA/cm^2^ 29,30, which were still lower than the films UV irradiated for 100 minutes (7.95 MA/cm^2^). Obviously, the improved density and uniform element distribution in the precursor films were the primary reasons for the enhanced J_c_ of YBCO films.

## Conclusion

In this study, high-performance YBCO films were produced by a novel DUV assisted solution deposition method. The acrylic acidic group sensitive to 254 nm UV light was used in the YBCO precursor solution, and resulted in the low-temperature decomposition of gel films exposed to the UV light. The UV irradiation prevented Cu aggregation and increased the film density, resulting in the enhanced crystallinity and superconductivity of the YBCO films. For the UV irradiated films, BYF, BaOF, and YOF, CuO formed as intermediate phases, which were finally converted to the YBCO phase when heated to 785 °C. Using a F/Ba = 2 solution with a metallic ion concentration of 0.75 mol/L, YBCO films with J_c_ as high as 8 MA/cm^2^ were obtained on the LaAlO_3_ substrates.

## Methods

### Preparation

A YBCO solution with a metallic ion ratio of Y:Ba:Cu = 1:2:3 was synthesized by mixing a Y-Ba-Cu solution and a Ba-TFA solution. 0.23 g of yttrium acetate, 0.28 g of barium acetate and 0.5 g of copper acetate were dissolved in a mixture solution of acrylic acid and methanol. The solution was stirred at 40 °C to produce the Y-Ba-Cu solution. The Ba-TFA solution was prepared by dissolving Ba-TFA gel in methanol, in which the Ba-TFA gel was synthesized as follows: 0.14 g of barium acetate was dissolved in a mixture solution of deionized water and trifluoroacetic acid (TFA) at room temperature, and then the mixed solution was evaporated in a vacuum oven at 75 °C for several hours to obtain a transparent glassy residue, namely the Ba-TFA gel. The YBCO solution was ready after the Y-Ba-Cu solution and Ba-TFA solution were mixed. The total metallic ion concentration of the YBCO precursor solution was maintained at 0.75 mol/L by regulating the amount of the methanol.

The YBCO precursor solution was coated on the LaAlO_3_ substrates by the dip-coating method. The coated gel films were air dried at 130 °C for 10 minutes to evaporate the methanol and excessive acrylic acid. The dried films were then placed on a hot plate at 150 °C and irradiated by a UV lamp with wavelengths of 253.7 nm (90%) and 184.9 nm (10%). The spectral distribution of the low-pressure mercury UV lamp is shown in Figure S4. The coating-drying-irradiation was repeated to build up the film thickness. After irradiation, photon-activated precursor films were obtained. The UV-irradiated films were then annealed at 785 °C under a mixture gas of humidified nitrogen and oxygen for 2 hours. Oxygen during the annealing process was maintained at a partial pressure of 500 ppm. After annealing, the films were post-annealed at 450 °C in oxygen for 2 hours. Finally, the films were furnace cooled to room temperature, and the YBCO superconducting films were produced. A sample without UV irradiation (the non-UV irradiated sample) and a reference sample were also prepared. The reference sample was prepared by a traditional solution method. The preparation processes of the non-UV irradiated sample and the reference sample are set forth in the supplementary Information section of this paper.

### Characterization

The UV-visible absorption spectra of the solutions and coated gel films were determined using a UV-Vis spectrometer. The films irradiated by the UV light were examined by a NieoletNexus 470 Fourier Transform Infrared Spectrometer. The surface morphology of the films was observed by a JEM-6700F scanning electron microscopy (SEM), and the thickness was confirmed by observation of the cross-section SEM image. The orientation, texture and the phase structures of the YBCO films were investigated by a 7000S-type X-ray diffractometer (XRD) and a high-resolution X-ray diffractometer (Rigaku martlab). The film was also characterized by a FEI Tecnai electron microscope. A multi-function Vibrating Sample Magnetometer (VersaLab-VSM, Quantum Design) was used to investigate the magnetization behaviors, and the sizes of samples were maintained at 2.1~2.5 mm × 2.1~2.5 mm. The J_c_ values of the films related to the magnetic strength (H) were calculated from the M-H curves according to the Bean Model. The obtained YBCO superconducting film was patterned to be a micro-bridge with a width of 150 μm. The V-I curve of the YBCO film UV-irradiated for 100 minutes was measured using the micro-bridge at 77 K to determine J_c_. Measurements of resistance versus temperature were taken at the temperature of liquid nitrogen using a standard four-probe method. The films were analyzed using Thermo Fisher X-ray photoelectron spectroscopy (XPS). A standard X-ray source, 15 kV, 150 W, Al Kα (1486.6 eV), was used for measurements. Survey and multiregion spectra were recorded at the Y 3d, Ba 3d, Ba 4d, Cu 2p, O 1 s, F 1 s, and C 1 s photoelectron peaks. A depth profiling was conducted using a 2 KeV Ar^+^ bombardment at a current density of 2 μA/cm^2^. A crater of 2 mm diameter was examined.

## Additional Information

**How to cite this article**: Chen, Y. *et al*. High Critical Current Density of YBa_2_Cu_3_O_7−x_ Superconducting Films Prepared through a DUV-assisted Solution Deposition Process. *Sci. Rep.* **6**, 38257; doi: 10.1038/srep38257 (2016).

**Publisher’s note:** Springer Nature remains neutral with regard to jurisdictional claims in published maps and institutional affiliations.

## Supplementary Material

Supplementary Information
