# Supplementary material for: High Critical Current Density of YBa2Cu3O7−x Superconducting Films Prepared through a DUV-assisted Solution Deposition Process
Source: Sci Rep. 2016 Dec 1;6:38257. doi: 10.1038/srep38257 (PMC5131298; doi:10.1038/srep38257)
Supplement: Supplementary Information [file srep38257-s1.pdf]

Supplementary information

High Critical Current Density of $YBa_2Cu_3O_{7-x}$ Superconducting Films Prepared through a DUV-assisted Solution Deposition Process

Yuanqing Chen[1*], Weibai Bian[1], Wenhuan Huang[2], Xinni Tang[1], Gaoyang Zhao[1], Lingwei Li[1], Na Li[1], Wen Huo[1], Jiqiang Jia[1] & Caiyin You[1]

[1] School of Materials Science and Technology, Xi'an University of Technology, Xi'an, Shaanxi 710048, China.

2 College of Chemistry & Chemical Engineering, Shaanxi University of Science & Technology, Xi'an, Shaanxi, 710021, China.

Corresponding Author: Yuanqing Chen (Dr.)

Email: chenyq@xaut.edu.cn (Yuanqing Chen)

Supplementary Figure S1

Schematic diagram of the UV-irradiation process

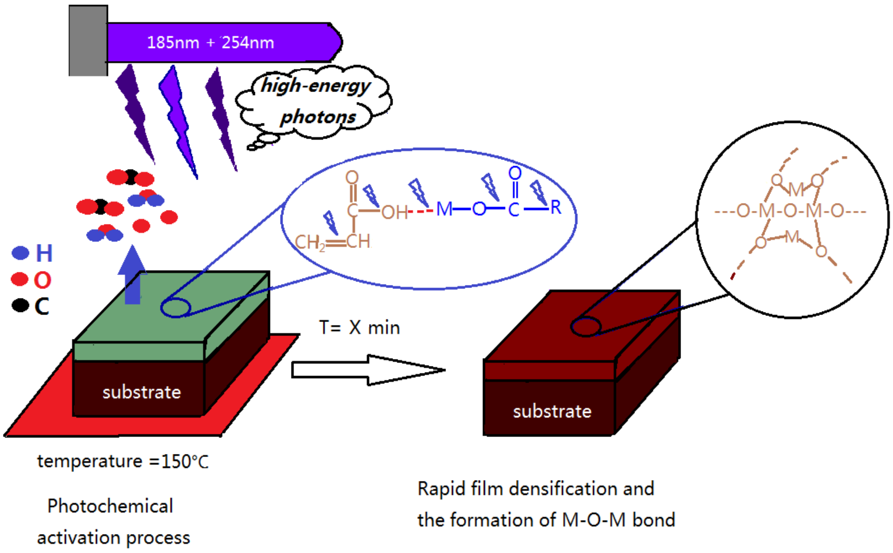

Supplementary Figure S2

(a) Depth profiling of the non-UV irradiated sample; (b) The C1s spectrum of the non-UV irradiated sample;

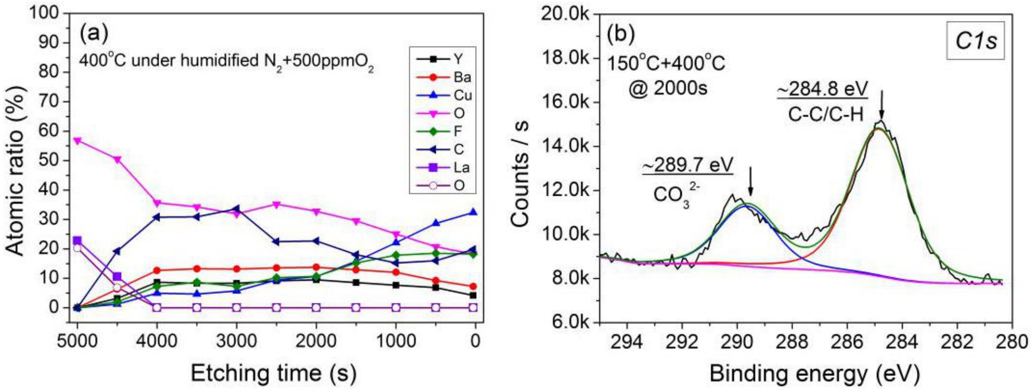

The non-UV irradiated sample was prepared as follows: 1) the coated gel film was derived from the YBCO precursor solution with F/Ba=2 (the ion concentration is 0.75mol/L) and heated at 150℃in air for 100 minutes, and then the coating-drying process was repeated to build up the film thickness; and 2) the dried film was heated to 400℃ under humidified N_2 with 500ppm O_2. After maintaining at 400℃ for 15 minutes, the sample was produced. Except the UV irradiation, the preparation process of the non-UV irradiated sample was same as the UV irradiated precursor film.

Depth profiling in Figure S2(a) indicated that the Y, Ba ions were uniformly distributed in the film. However, the Cu and F elements were not uniformly distributed in the film, and large amount of carbon residue was detected. In the C1s spectrum, two peaks located at 284.8eV and 289.7eV were detected. The peak at 289.7eV corresponded to the carbonate (CO_3^{2-}). In other words, in addition to residual carbon bonds, an abundance of carbonates existed inside the film because of the $BaCO_3$ phase formed during the decomposition of the gel film. XPS revealed that inside the film, the F/Ba was even less than 1 in some areas, indicating that in addition to BaF_2 or BaOF, $BaCO_3$ was formed inside the film, which was consistent with the results revealed by the C1s spectrum.

Supplementary Figure S3

(a) The depth profiling of the reference sample; (b) the low-angle XRD of the reference sample.

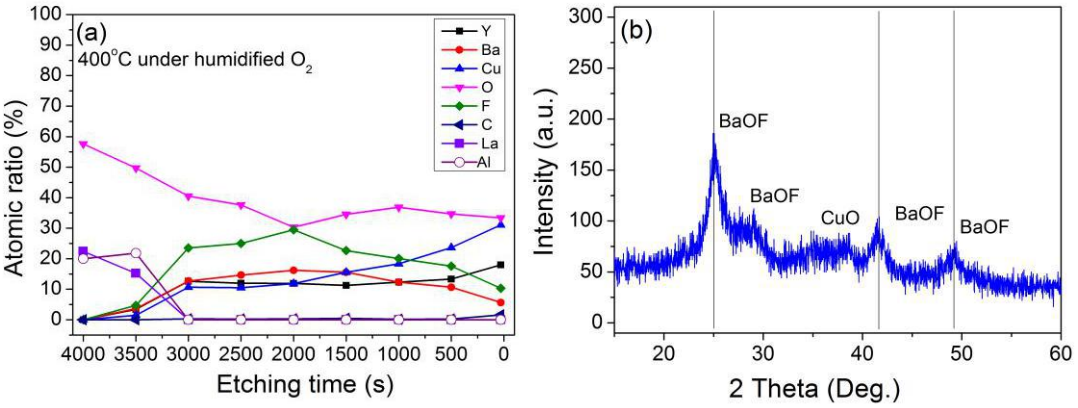

The reference sample was prepared as follows: 1) the coated gel film derived from the YBCO precursor solution with F/Ba=2 (the ion concentration is 1.5mol/L) was heated at 100°C in the air for 10 minutes; and 2) the dried film was heated to 400°C under humidified O_2. After being maintained at 400℃ for 15 minutes, the sample was obtained. This pyrolysis process was same as the traditional TFA route used for fabrication of the YBCO film [1].

The depth profiling in Figure S3(a) indicated that the Y, Ba, Cu and F elements were not uniformly distributed, and that the F/Ba was between 1.5-2. Thus, the Ba^{2+} existed in the form of BaF_2/BaOF, unlike when a solution with F/Ba>2 was used [1]. The XRD in Figure S3(b) confirmed this conclusion. The XRD also indicated that no $BaCO_3$ phase was detected in reference sample.

Supplementary Figure S4

Spectral distribution of the high-pressure mercury UV lamp.

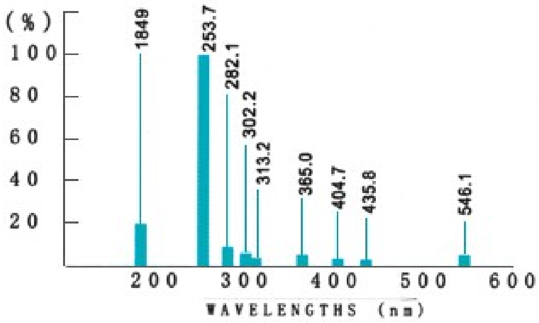

References:

[1] Yuanqing Chen, Xiaoru Yin, Yi Feng, Weibai Bian, Mengjuan Li, Jinfen Niu, High-efficiency preparation of high-quality YBCO superconducting films using an ultralow-fluorine sol–gel method, Journal of Sol-gel Science and Technology 74, 249–255 (2015).
